# Nurses’ perspectives on obtaining best possible medication histories in a virtual care setting: a qualitative study

**DOI:** 10.1186/s12913-026-15504-z

**Published:** 2026-09-07

**Authors:** Vickie Zhiyan Zhang, Melissa Pham, Adrian Boscolo, Katrina Armour, Martina Francis

**Affiliations:** 1 Sydney Local Health District Virtual Hospital, Sydney Local Health District, Sydney, New South Wales Australia; 2 Digital Health and Innovation, Sydney Local Health District, Sydney, New South Wales Australia; 3 Department of Pharmacy, Royal Prince Alfred Hospital, Sydney Local Health District, Sydney, New South Wales Australia; 4 School of Pharmacy, Faculty of Medicine and Health, The University of Sydney, Camperdown, New South Wales Australia

**Keywords:** Best possible medication history, Medication reconciliation, Virtual care, Nurses’ role, Medication safety, Interdisciplinary collaboration

## Abstract

**Background:**

Medication discrepancies during transitions of care are a major and preventable source of medication-related harm. A Best Possible Medication History (BPMH) is a critical first step in medication reconciliation and risk mitigation. While nurses are increasingly recognised as contributors to medication safety, limited evidence has explored their experiences obtaining BPMHs within a virtual care setting. This study aims to explore nurses’ perspectives on obtaining BPMHs for patients in a virtual care setting, including perceived enablers, challenges, and implications for practice optimisation.

**Methods:**

A qualitative study was undertaken at a metropolitan virtual hospital in Sydney, Australia. Semi-structured interviews were conducted between March and July 2025 with accredited virtual care nurses and midwives who had independently performed BPMHs. Interview questions were informed by selected domains from the Theoretical Domains Framework, and data were analysed inductively using Braun and Clarke’s six-phase thematic analysis.

**Results:**

Fifteen of twenty-one eligible nurses participated. Participants represented a highly experienced nursing workforce with a mean of 18 years of nursing experience and 4 years of virtual care experience. Three themes were identified: (1) Collaboration and workflow integration, (2) Education and feedback, and (3) Challenges. Nurses initially expressed uncertainty regarding role boundaries and accountability but progressively viewed BPMH as a valuable and relevant extension of nursing practice. Practical scenario-based learning and timely feedback were perceived as critical to skill development and sustainable workflow integration. Key challenges included patient acuity, digital literacy, limited visual assessment during consultations, fragmented communication, workload pressures, and limited protected time for comprehensive BPMH activities.

**Conclusions:**

Nurse-led BPMH is feasible in a virtual care setting when supported by multidisciplinary governance, structured education, and integrated support systems. This study provides novel insights into a scalable nurse-led BPMH workforce model within virtual care.

**Supplementary Information:**

The online version contains supplementary material available at https://doi.org/10.1186/s12913-026-15504-z.

## Background

Transitions of care, such as hospital admission and discharge, are consistently identified as vulnerable points for medication-related harm along the healthcare continuum [1, 2]. Globally, medication-related errors are a major contributor to patient harm during these transitions. A systemic review found that a median of 53% adults and older patients experienced at least one medication error following hospital discharge, 50% had medication discrepancies, and 19% experienced an adverse drug event [3]. Across Australian healthcare settings, medication errors and adverse drug events affect approximately 30–80% of patients [4]. Among medication errors and discrepancies reported, medication omissions are the most common, with up to 75% considered clinically significant [4, 5]. Medication-related problems also account for 15.3% of unplanned hospital readmissions, more than half of which are considered preventable [6]. These findings highlight the clinical and health-system burden associated with medication-related harm.

The World Health Organization (WHO) has identified transitions of care as a priority for intervention and has advocated global strategies to reduce medication-related harm [7, 8]. Medication reconciliation is a formal, structured process in which the medications a patient is taking are compared with current medication orders at transitions of care, with discrepancies identified, resolved and communicated to the patient and relevant care providers [9–11]. The WHO and Australian Commission on Safety and Quality in Health Care (ACSQHC) recommend a formal, structured four-step medication reconciliation process: obtaining a Best Possible Medication History (BPMH), comprising of a thorough history of all regular medications prescribed and non-prescribed, and confirmed with at least one other reliable source; reconciling the BPMH against prescribed medications and updating the medication list at every step of care; and finally, supplying accurate medication information to patients, and/or carers, and subsequent healthcare providers [9, 11]. Medication reconciliation is a key strategy to support medication continuity and reduce medication discrepancies during care transitions. Evidence suggests that medication reconciliation, when initiated as early as possible during an episode of care, can reduce medication errors at transition points [9–11]. Robust evidence demonstrates that structured medication reconciliation can reduce medication discrepancies by up to 70% and significantly reduce post-discharge healthcare utilisation [12, 13].

Medication reconciliation roles vary across health services internationally and are commonly undertaken by pharmacists and medical practitioners in acute hospital settings [14]. However, workforce constraints, increasing service demands and a growing emphasis on medication safety have strengthened recognition of medication reconciliation as a shared, interprofessional responsibility. Nurses, as the largest and most consistently patient-facing professional group, are well positioned to contribute to this process when supported by appropriate education, training and governance structures [15, 16]. One randomised controlled trial found that hospital nurses trained in a structured method for medication reconciliation had similar number of clinically relevant medication discrepancies to clinical pharmacists [17], while another study showed that nurse–pharmacist collaborative models may be efficient and cost-effective in preventing potential adverse drug reactions [17, 18].

Despite this, nursing involvement in medication reconciliation remains variable. Reported barriers include unclear role definitions, professional boundary concerns, and inconsistent access to training and practice guidance. In acute hospital settings, nurses have described their role in medication reconciliation as minor compared with that of pharmacists and physicians [14, 16, 19]. These factors have contributed to inconsistent attitudes toward documenting and reporting discharge medication discrepancies. Some nurses have also reported reluctance to perform discharge medication reconciliation due to concerns about professional accountability and practice legalities, perceiving these activities as outside their scope of practice [14].

In response to global medication safety governance initiatives, and workforce constraints, Australia’s first virtual hospital implemented a nurse-led BPMH practice in April 2024. Embedded into virtual care workflows during scheduled initial and follow-up nursing assessments, this practice enabled accredited nurses to collect, verify and document patients’ medication histories as the essential component of medication reconciliation. Reconciliation of the BPMH and resolution of clinically significant discrepancies remained a multidisciplinary process, involving medical and pharmacy input where required. The aim of the study was to explore nurses’ perspectives on obtaining best possible medication histories in a virtual care setting, including perceived enablers, challenges, and implications for practice optimisation.

## Methods

### Study design

This qualitative descriptive study used semi-structured interviews to explore nurses’ experiences and perspectives of a nurse-led BPMH practice within a virtual care setting, including perceived enablers and challenges. The research team included multidisciplinary clinicians and investigators with expertise in medication safety, nursing practice, pharmacy, medicine and virtual care. Reporting was guided by the Consolidated Criteria for Reporting Qualitative Research (COREQ) checklist.

### Study setting and recruitment

The study was conducted at a virtual hospital in Sydney, New South Wales, Sydney Local Health District Virtual Hospital, (Sydney Virtual). Sydney Virtual was the first virtual hospital in Australia, established in early 2020 to support capacity challenges forecast and improve patients’ healthcare experience through the delivery of scalable, digitally enabled hospital-level care to patients in the community [20]. Virtual clinical care is delivered through video consultations and supported by multiple modalities including synchronous and asynchronous remote monitoring using smart and wearable devices [20]. Semi-structured interviews were undertaken between March and July 2025.

Participants were eligible if they: (1) were registered nurses or midwives delivering virtual care services; (2) had completed the mandatory BPMH education and clinical skills assessment; and (3) had independently performed at least one BPMH in routine clinical practice following implementation of the practice in April 2024.

Given the novelty of the virtual hospital model and the relatively small eligible nursing workforce, convenience sampling was used to maximise participation. Recruitment information was disseminated through nursing staff email distribution list, bulletin board flyers, and nursing handover and huddle sessions. Up to three recruitment reminders were distributed across these channels to increase staff awareness and optimise participation.

Interested participants were provided with the Participant Information Sheet and given the opportunity to ask questions before providing written informed consent. Participants were informed that they could withdraw from the study at any time. Data collected before withdrawal were retained and included in the analysis, in accordance with the Participant Information Sheet and approved ethics protocol.

### Data collection

The interview guide was developed specifically for this study and informed by selected domains from the Theoretical Domains Framework (TDF) and relevant literature [21]. The TDF is an integrative theoretical framework comprising of 14 domains refined though empirical validation [21]. The TDF was used as a sensitising framework, with minor adaptations made to align with the study aim and implementation context. The interview guide is provided as a supplementary file. Eight domains were explored: Knowledge; Professional Role and Identity; Emotion; Education; Beliefs about Capabilities; Environmental context and Resources; Feedback and Support; and Goals. Open-ended questioning and additional prompts enabled identification of influences beyond these domains and facilitated deeper exploration of participant responses.

The interview guide was piloted with two volunteer nurses who were not included as study participants. Feedback was obtained through interviewer observations and volunteer feedback. This process was used to identify gaps, clarify wording and refine the questions before formal data collection commenced.

One-on-one semi-structured interviews were conducted in person or via Microsoft Teams, according to participants’ preference. Interviews ranged from 30 to 60 minutes. At the beginning of each interview, non-identifiable demographic information was collected. Interviews were conducted by two study investigators, AB and MF, who had no prior direct professional or supervisory relationship with participants. With participant consent, interviews were recorded using Microsoft Teams, transcribed verbatim, de-identified and proofread for content accuracy. Recruitment and data collection continued throughout the study period. Data sufficiency was considered using principle of information power, and the final dataset of 15 participant interviews was considered sufficiently rich and relevant to support robust reflexive thematic analysis and address the study aim [22].

Participants received an AUD $10 café voucher at the conclusion of the interview as a token of appreciation for their time.

### Data analysis

De-identified transcripts were analysed independently by study investigators, MP and KA, using Braun and Clarke’s six-phase thematic analysis framework [21]. The analysis involved familiarisation with the data, generation of initial codes, development of preliminary themes, review and refinement of themes, theme definition and final reporting.

Initial coding was conducted inductively to enable themes to be generated from participants’ accounts. Initial codes and preliminary themes were compared and discussed between the investigators, then consolidated into a shared coding framework. Two additional study investigators, VZ and MF, independently reviewed the coding framework and preliminary themes to refine definitions and support analytical rigour, consistency and consensus. Final themes were then considered in relation to relevant domains to support interpretation rather than pre-determined by the framework.

### Ethical considerations

Ethics approval was obtained from the Sydney Local Health District Human Research Ethics Committee (X25-0023 & 2025/ETH00215). The study was conducted in accordance with the National Statement on Ethical Conduct in Human Research (2023) issued by the National Health and Medical Research Council, Australian Research Council and Universities Australia. All data presented in this study have been de-identified.

The AUD $10 café voucher reimbursement was outlined in the Participant Information Sheet, Participant Consent Form, Ethics Protocol and study advertising materials to ensure transparency.

Study investigators who met the study eligibility criteria participated in the role of participants only in accordance with the approved conflict-of-interest management process.

## Results

### Participant characteristics

A total of 21 virtual care nurses met the eligibility criteria for participation, and 16 consented to be interviewed. One participant was subsequently excluded after reporting they had not completed a BPMH independently, resulting in a final sample of 15 participants.

Participants represented a highly experienced nursing workforce, with a mean of 18 years of nursing experience and 4 years and 2 months of virtual care experience. Most participants were employed full-time (*n* = 11), and the remaining worked part-time (*n* = 4). Approximately 60% of participants (*n* = 9) reported prior experience in collecting medication histories. However, this experience was generally limited in scope and breadth and did not reflect the level of responsibility required within the current nurse-led BPMH practice.

### Themes

**Fig. 1:**
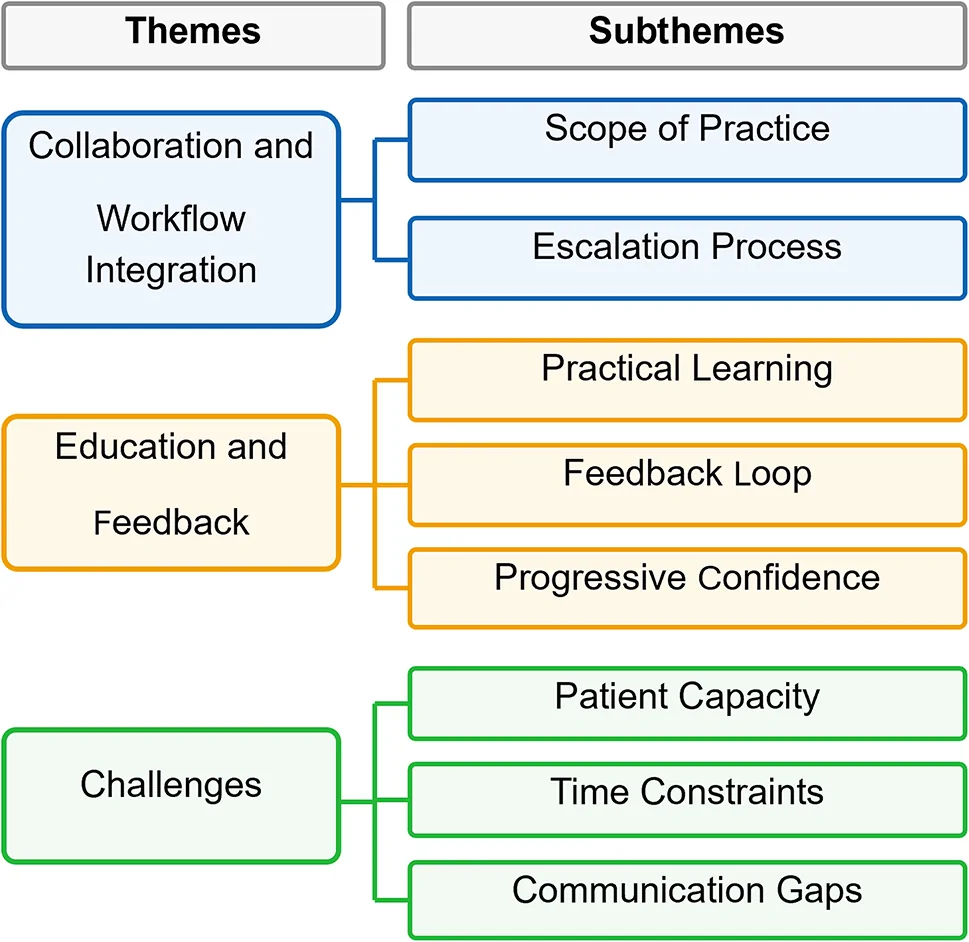
Overview of themes and subthemes

Thematic analysis revealed three key overarching themes, each comprised of multiple subthemes: (1) Collaboration and workflow integration; (2) Education and feedback; and (3) Challenges, as illustrated in Fig. 1.

## Collaboration and workflow integration

Following inductive development, this theme was interpreted as relating to the domains of Professional Role and Identity, Social Influences and Goals, and comprised of two subthemes.

### Scope of practice

Participants initially hesitated about whether obtaining a BPMH aligned with their professional role and scope of practice, which contributed to early uncertainty about adopting the new practice. Some expressed concern based on prior experience, where BPMH and medication reconciliation were typically associated with pharmacists and physicians. As a result, participants were initially apprehensive about taking on this responsibility and integrating the task into their existing workflow.These concerns were reflected in statements indicating that nurses initially believed BPMH was the responsibility of doctors or pharmacists.

*At the very beginning*,* I was hesitant because as a registered nurse*,* when I used to work in a hospital*,* this is not part of our duty of care*. (P6)

Acceptance of practice increased when BPMH was positioned as a collaborative responsibility, with participants willing to take greater ownership while remaining supported by pharmacy and medical disciplines.

*We are happy to help. We want to take ownership of it*,* but we want to be co-ownership. We don’t want to be [in] complete ownership of the BPMH*,* if that makes sense. If not pharmacy*,* it could be co-shared with the doctors too because I do understand that there are doctors [and] pharmacists. It should be shared with everyone rather than just being*,* you are responsible…* (P7).

As nurses gained experience and confidence, many reported a shift in perspectives regarding BPMH as a valuable extension of holistic nursing practice that supports safer care delivery with additional medication-related clinical context provided.

*I feel that it is part of our role because it gives us a total care for the patient*,* not just the physical care but in regard to medications*. (P5)

The virtual care environment was also perceived as supporting deeper engagement with tasks, facilitated by easier access to digital systems and the opportunity for protected one-to-one patient interaction.

*Specifically in virtual care*,* we do have the capacity to be more involved*. (P11)

Several participants pointed out downstream clinical benefits, noting that early BPMH facilitated safer prescribing and discharge processes.

*They [BPMH] actually save the whole team lots of time… it makes it easier for the doctors when they do the discharge*. (P14)

### Escalation process

Consistent with participants’ preferences for shared responsibility and collaborative practice, clear escalation processes and having multiple sources of support are desired. They expressed that knowing when and how to escalate BPMH-related issues, enabled them to build their confidence, safely integrate this task into their workflow, and facilitated sound BPMH practice. Nurses described clear escalation protocol typically began with peer discussion, followed by escalation to the Nurse Unit Manager or Clinical Nurse Educator and further medication review with the pharmacist or medical team as required. This tiered approach enabled nurses to obtain the BPMH within their scope while ensuring that complex or unresolved concerns were reviewed by the appropriate pharmacist or medical clinicians.

*Having it standardised more into practice - the escalation pathways*,* has been helpful.* (P11)

## Education and feedback

This theme related primarily to the domains of Knowledge, Skills, Beliefs about Capabilities, and comprised of three subthemes.

### Practical learning

Participants expressed a strong preference for practical learning. While theory and online resources were viewed positively, they emphasised the benefit of face-to-face, scenario-based training to consolidate learning. They expressed that observing and practicing how the BPMH process should be conducted supported their confidence in applying medication-related knowledge to practical clinical situations.

*The very best one was the pharmacist actually running the BPMH education… Good face to face value and very straightforward than a lot on HETI or other online courses.* (P7)

*Scenario type of thing on how it gets done. I only learn from scenarios that I’ve seen. So visually seeing this is what you do or trying it out when I’m practicing it with somebody*,* then it goes in easier.* (P5)

### Feedback loop

Prompt, real-time performance feedback from the virtual care pharmacist and senior nurses helped participants refine their BPMH practice after formal education and competency assessment. This approach enabled them to refine their interviewing, verification and clinical documentation skills within BPMH practice as an ongoing learning mechanism rather than solely as performance monitoring. Constructive feedback and recognition also reinforced nurses’ sense of being valued and acknowledged for their roles in medication safety. Accessible pharmacist support reduced nurses’ anxiety about overlooking discrepancies, reinforced safe practice, and supported participants’ confidence in independently undertaking BPMH.

*The pharmacist is always onsite and I think she does her checks on the background. When she checks those entry*,* she will be able to let us know which one did good*,* which one needs improvement and what needs to be done.* (P6)

*I would say she* [the virtual care pharmacist]*’s actually very good with her feedback. She’s very constructive*,* very straight to the point*,* not beating not going around circular communication*,* very straightforward. ‘You’ve done this well.’ ‘You just missed this one thing’*,* ‘maybe in the follow-up’*,* like prompt comments*,* ‘you can do this better’.* (P7)

### Progressive confidence

Participants described confidence and gradual skill growth developed progressively through a positive feedback loop, alongside clear communication and supportive integration of BPMH into clinical workflows. Consequently, participants reported that obtaining BPMHs was perceived to become easier with repeated practice.

*Since doing the BPMH*,* medication audit (results) has actually improved drastically…That was fed back to us during meeting or regularly when they do the audits. That was really good and also gets regular feedback as well when you’re doing the right thing.* (P2)

*I feel pretty confident in performing it*,* but it’s something that takes practice.* (P10)

## Challenges

This theme related primarily to the domains of Emotion and Environmental Context and Resources and comprised of three subthemes.

### Patient capacity

Patients who were acutely unwell, fatigued following hospital discharge, unable to recall medication details or had limited understanding of their medications were reported less able to engage in BPMH interview. Limited digital proficiency in using smart devices for video consultations compounded these challenges by restricting nurses’ ability to visually inspect and verify their medications.

*As I said before*,* we’re trying to do this in the initial call. If we can’t*,* we’ll do this in the follow-up call. But sometimes when patient got discharged from the hospital*,* they’re exhausted. They don’t have much energy or time to give us all this information*,* especially when people are not feeling well and we’re just bombarded with them with all these questions*,* all these details. It can cause a lot of tension and some reluctance and hesitance from their side.* (P6)

*The harder way*,* if the patient*,* they’re very forgetful*,* if they don’t have all their medication with the boxes and all the labels*,* we can’t really identify it correctly. So that makes it a bit trickier.* (P4)

*A lot of our patients that we’ve called*,* they haven’t been able to do the video because they’re quite a bit older. They don’t have a smartphone*,* things like that. So obviously we can’t… (P4)*.

### Time constraints

Time pressure arose from the time required to conduct a thorough BPMH, the subsequent verification required across multiple medication information sources and identification and resolution of any discrepancies. Contacting general practitioners or community pharmacies to obtain past prescribing and dispensing histories, awaiting responses and completing clinical documentation could extend the process beyond the initial assessments. Competing responsibilities for other patient cohorts, incoming calls and staffing resources were also reported to affect the timely and consistent completion of BPMH.

*It is time consuming sometimes when there are discrepancies between what patients are telling us and what’s been prescribed to them.* (P3)

*They’re thinking*,* what if we have more activity in the future? Do you think I really have time to spend extra ten*,* fifteen minutes or emailing the pharmacist or the GP and then waiting? What if you miss things? I think they’re worried about that.* (P7)

### Communication gaps

Communication gaps also affected the continuity and completion of BPMH, particularly when required follow-up actions were not clearly documented or information was lost during clinical handovers.

*…because of the gaps sometimes with handover and documentation. Maybe something wasn’t handed over properly and then some things get missed. And then that BPMH is never actually properly completed.* (P8)

## Discussion

This qualitative study identified key enablers and barriers to nurses obtaining BPMHs within a virtual hospital. Clear scope of practice, practical education, timely feedback and accessible escalation supported integration into nursing practice, while patient capacity, time and workload pressure, and communication gaps constrained completion. These influences were reflected across three overarching themes: *Collaboration and workflow integration*, *Education and feedback*, and *Challenges*. Collectively, these themes show how nurses navigated evolving role boundaries, developed professional competence and managed practical complexities of obtaining BPMH within virtual care. These findings highlight the key influences during implementation, thereby directly addressing the study aim.

### Collaboration and workflow integration

Participants revealed initial uncertainty between traditional professional boundaries and emerging expectations for shared responsibility in obtaining BPMHs. Early in implementation, obtaining BPMHs was perceived as outside of the traditional nursing scope of practice, consistent with previous literature describing uncertainty and hesitancy of nurses when undertaking medication-related activities historically performed by pharmacists or medical practitioners [14, 16, 19]. Over time, however, participants progressively viewed BPMH as clinically appropriate and relevant to nursing practice, particularly in relation to patient safety and continuity of multidisciplinary care.

This shift in perspective appeared to develop gradually through repeated practice exposure, organisational support and integration of BPMH into routine clinical workflows. Importantly, clearly defined escalation pathways emerged as a critical enabler. Access to pharmacist and senior nursing support reduced participants’ fear of making mistakes or raising concerns, facilitating timely intervention, shared accountability, and confidence in practice. Nurses’ willingness to engage in BPMH was strongly influenced by the presence of a non-punitive environment in which expert support was readily accessible.

These findings indicate that sustainable nurse-led BPMH practice relies not solely on individual clinician capability but also on strong interdisciplinary collaboration, accessible support structures and shared governance. Participants’ evolving professional ownership, confidence and perceptions of BPMH as a meaningful patient-safety activity also suggest motivational determinants beyond capability and opportunity alone.

The findings align with Feldman et al. [18], who demonstrated that nurse–pharmacist collaboration can identify unintended medication discrepancies and prevent potential harm, while remaining efficient and cost-effective. The present findings extend this evidence by showing how such collaboration is experienced by nurses in practice. Participants did not seek full independent ownership of BPMH; similarly, they valued co-ownership, escalation pathways and pharmacist oversight. This reinforces the view that nurse-led BPMH is most acceptable when positioned as a collaborative medication safety activity, rather than as task substitution or professional role displacement.

Similarly, the findings support emerging literature that positions medication reconciliation as an interprofessional responsibility rather than the sole domain of pharmacists or medical practitioners. Latimer et al. [14] found that acute care nurses in Australian hospital settings recognised their contribution to medication reconciliation through “chasing, checking and educating,” yet were constrained by unclear guidance, insufficient training and workload pressures. Comparable perceptions were present, where study participants initially viewed BPMH as outside traditional nursing responsibilities, particularly because it had historically been pharmacist-led. However, unlike the findings reported by Latimer et al. [14], virtual care nurses described a clearer progression from uncertainty to professional ownership. This suggests that structured accreditation, repeated clinical exposure and accessible pharmacist support facilitate sustained role integration. However, future implementation may benefit from greater protected clinical time to support comprehensive history taking, medication verification and follow-up.

### Education and feedback

Although in-service education and written resources were regarded as useful foundations for practice, participants consistently emphasised that BPMH capability was most meaningful through practical learning and clinical application. This highlights the limitations of passive teaching approaches for complex medication-related tasks. Pharmacist-led, face-to-face education and scenario-based learning were regarded as particularly effective and engaging, supporting the translation of theoretical concepts into clinical practice.

The learning trajectory described by participants reinforces BPMH as a progressively acquired clinical skill. Early difficulties or limited engagement should therefore not be interpreted solely as resistance or a barrier to practice adoption, but as part of a normal learning curve requiring reinforcement, protected time, and ongoing support following accreditation. Consistent feedback also functioned as a key mechanism for transparency, continuous improvement and professional validation. Real-time feedback, from the pharmacist and senior nursing staff was not only corrective but also affirming, strengthening nurses’ sense of contribution and professional value. Importantly, feedback appeared to support sustainable behavioural change beyond the initial implementation phase. Without visible feedback, the nurse-led BPMH process risked being perceived as an additional task rather than a meaningful and valued component of clinical care.

The importance of structured education in this study is also consistent with New South Wales Clinical Excellence Commission (CEC) guidance [23]. The CEC emphasises that a BPMH includes obtaining a medication history, and subsequently verifying it with at least one other reliable source. This accounts for the first two crucial steps of medication reconciliation. The CEC nursing and midwifery education pilot similarly found that policy development, organisational support and nurse-specific training were necessary to support nurses undertaking medication-related tasks. Participants in this study echoed these requirements, but placed particular emphasis on practical, scenario-based and pharmacist-led face-to-face training, suggesting that online learnings alone may be insufficient for complex clinical skill development.

These findings have informed several practice translation strategies, including embedding BPMH education and competency assessment into routine nurse orientation to support consistent capacity development; developing a co-designed BPMH lanyard card as an accessible quick reference guide, particularly when timely in-person support may not be immediately available; and reviewing BPMH performance at monthly nursing ward meetings to address staff queries, close feedback loops and support continuous practice improvement.

### Challenges

Participants identified several challenges pertinent to conducting BPMHs in virtual care. Patient-related factors, including their health status, readiness to engage, digital literacy and understanding of their medications may influence the completeness and accuracy of medication information obtained as the primary source. Reliance on video consultations may further compound these challenges, particularly when visual quality was poor or unavailable, limiting clinicians’ ability to visualise and verify medications and increasing reliance on secondary information sources such as general practitioners, community pharmacies and existing clinical documentation. Time constraints and workload pressure also emerged as dominant challenges. Obtaining a BPMH requires a high level of information accuracy, verification and follow-up, while competing with other patient assessments required during initial virtual consultations. The BPMH practice was embedded within these clinical duties rather than allocated with separately protected time.

Future practice should consider strategies to improve patient digital literacy, strengthen use of existing digital support resources and engage designated digital support staff to enhance patient’s readiness for virtual consultations. Providing patients with advanced notice and clear instructions prior to virtual BPMH interviews may reduce engagement barriers and improve efficiency. Further efforts should also advocate for protected clinical time within routine patient review workflows to reduce avoidable task deferral, support more consistent completion, and strengthen the sustainability of the BPMH practice.

The challenges identified mirror previous reports. Al-Hashar et al. [24] found that healthcare professionals recognised the value of medication reconciliation but differed in their perceptions of professional responsibility, with barriers including unreliable medication sources, patient knowledge gaps, poor coordination and time limitations. Some of these barriers may be more pronounced in the virtual care setting than conventional face-to-face settings. Virtual care nurses may be unable to physically inspect medication packaging, prescriptions or dose administration aids and increase reliance on patient-reported information, variable patient digital literacy and fragmented external information from general practitioners and community pharmacies. In conventional settings, these materials may be more readily available for direct verification. Conversely, virtual care may facilitate focused one-to-one interaction and readily access to digital clinical systems. These contextual differences should be considered when interpreting the transferability of the findings across various care settings.

Pharmacist-led BPMH studies continue to show superior completeness and accuracy compared with usual ward-based collection. For example, Mengato et al. [25] reported markedly fewer omissions when BPMHs were collected by pharmacists compared with standard ward staff processes. The present study should therefore not be interpreted as evidence that nurse-led BPMH replaces pharmacist expertise, but as evidence that trained nurses can expand medication safety capacity in settings where pharmacist availability is limited, provided there is robust governance, escalation, audit, and feedback.

### Strengths

A key strength of this study is the exploration of nurses’ experience of obtaining BPMH within real-world virtual care delivery, rather than in a pilot or simulated setting. This strengthens practical relevance of the findings and supports their transferability to other digitally enabled and remote care models.

By situating nurses’ experiences within an established virtual hospital and its existing service models, this study provides implementation-relevant insights into an important yet under-researched area of virtual medication safety practices. The findings demonstrate how professional roles, education and feedback, workflow demands and digital models of care interact in routine virtual care delivery. The adoption of the TDF as a sensitising framework provided an evidence-based and applicable theoretical lens for interpreting implementation-related findings. The inductive analytic approach nevertheless allowed participants’ accounts to shape theme development rather than constraining findings to selected TDF domains incorporated into the interview guide. Together, these findings contribute practical evidence for an emerging multidisciplinary approach to medication safety that has traditionally associated with pharmacy and medical disciplines, but increasingly relevant to broader multidisciplinary care.

Another strength is the robust interprofessional and collaborative lens adopted throughout the study. Investigators from nursing, pharmacy, and medical disciplines contributed to study design, data collection and analysis. This multidisciplinary approach strengthened analytic depth, reduced the risk of single-discipline interpretation, and enhanced the credibility and interpretive rigour of the study findings.

This study is also highly relevant to contemporary healthcare priorities. As NSW Health continues to invest in virtual care, supporting increasingly complex patient cohorts through digitally enabled models, the nurse-led BPMH practice described in this study offers a replicable example for embedding medication safety practice within virtual care delivery. Its alignment with medication safety standards and digital health transformation priorities further strengthens its relevance for health services navigating concurrent clinical, workforce and digital reform agendas.

### Limitations and future directions

Several limitations should be acknowledged. First, the study was conducted at a single metropolitan virtual hospital in New South Wales, which may limit transferability to settings with varying organisational frameworks, digital maturity and adoption or workforce models. Nonetheless, the detailed contextual description of the implementation setting may assist readers in assessing relevance and applicability of the findings to other services.

Participants were recruited from a cohort of virtual care nurses who had completed BPMH education and accreditation. This may have introduced selection bias toward clinicians who were more confident, experienced, or engaged in practice. The perspectives of nurses who did not meet eligibility criteria were not captured.

The study interview guide was informed by eight domains from the TDF, which were selected for their relevance to study aim and implementation context, rather than designed to comprehensively examine all fourteen domains. This focused application supported contextually relevant exploration but may have limited the exploration of other determinants, particularly Optimism, Beliefs about Consequences and Intentions. Open-ended questioning enabled participants to raise influences beyond the selected focuses, and themes were developed inductively before TDF interpretation. Findings should therefore be viewed as an implementation-focused account in the study setting, rather than as a comprehensive assessment of all determinants. To support transparency and reproducibility, the adapted interview guide is provided in supplementary file.

Lastly, findings were based on self-reported experiences and may be subject to recall or social desirability bias in a professional context. Strategies to mitigate this included interviewer neutrality, assurances of voluntary participation and confidentiality, and the use of open-ended questioning with neutral probing prompts to encourage honest and reflective responses.

While this study focused on nurses’ qualitative experiences of obtaining BPMH in virtual care, future research may consider mixed-methods designs incorporating quantitative medication safety outcomes. These may include BPMH completion rate, medication discrepancy rates, preventable medication-related incidents, and hospital readmission rates to strengthen the evidence base for this model of practice. Future studies could examine motivational TDF domains more comprehensively, and explore patients' perspective on nurse-led BPMHs, which may also provide valuable insights.

## Conclusions

This study contributes novel implementation-focused evidence by exploring nurse-led BPMH practice within a virtual hospital in Australia. Nurses perceived the practice as a feasible and professionally relevant component of virtual care when supported by clear role expectations, structured and practical education, timely feedback, accessible multidisciplinary support and shared accountability. These findings support nurse-led BPMH as a collaborative medication safety practice that can broaden workforce participation while supported by pharmacy and medical oversight for medication review, reconciliation and clinically indicated interventions.

## Supplementary Information

Below is the link to the electronic supplementary material.

Supplementary Material 1

## Data Availability

Data supporting the finding of this study may be made available from the corresponding author upon review of reasonable request, and in accordance with the approval ethics protocol. The data are not publicly available due to information that could compromise the confidentiality and privacy of research participants.

## Abbreviations

WHO: World Health Organisation
ACSQHC: Australian Commission on Safety and Quality in Health Care
BPMH: Best Possible Medication History
CEC: Clinical Excellence Commission
COREQ: Consolidated Criteria for Reporting Qualitative Research
TDF: Theoretical Domains Framework
NSW: New South Wales
