# Supplementary material for: Nurses’ perspectives on obtaining best possible medication histories in a virtual care setting: a qualitative study
Source: BMC Health Serv Res. 2026 Sep 7;26:1320. doi: 10.1186/s12913-026-15504-z (PMC13621627; doi:10.1186/s12913-026-15504-z)
Supplement: Supplementary file 1 [file 12913_2026_15504_MOESM1_ESM.docx]

Supplementary Material- Interview Guide

**I. INTRODUCTION**

Hello, my name is [name of interviewer]. *Introduce self and other team members in the room.*

1. *Introduce the project*

The purpose of this study is to identify the challenges and enablers that nurses/ midwives encounter when performing Best Possibly Medication History (BPMH) during virtual care hospital admissions. Is this an appropriate time to interview you?

Yes	CONTINUE

No	SET UP A TIME LATER

2. *Explain the purpose of the interview*

We will be interviewing RNs within the team that have completed BPMH education and clinical skill assessment and performed at least one independent BPMH. We are really interested in learning more about your experiences and any benefits or challenges you have come across when obtaining BPMH from patients. We will use this information to inform strategies to better support you in this role in the future.

3. *Describe the audio recording and how we will assure confidentiality and answer any questions*

This interview will be approximately 30 minutes and will be audio recorded and transcribed for accuracy. Your responses will remain confidential and deidentified and securely stored in accordance with the SLHD Ethics approved guidelines.

If you need to contact me at any time after the interview to ask any questions, my contact details are *[my work email: X.X @health.nsw.gov.au].*

If desired, you will be given the opportunity to review the completed transcript prior to publication and analysis.

If, at any time, you feel that the questions are too sensitive, I would be happy to turn off the recorder during that portion of questioning. You may also skip any questions you wish during the interview.

Before we begin the interview and start recording, do you have any questions for me? *[Answer any questions]*

Demographic Information

**Professional Background**

**Clinical experience:**

- How many years have you been working as a nurse or midwife?

(e.g., <1 year, 1–5 years, 6–10 years, 11–15 years, 16+ years)"

- What, if any, prior experience do you have in collecting and documenting medication history?
- How long have you been working in this field?

**Working schedule**

- Are you currently working full-time, part-time or casual basis?
- On average, how many hours per week do you work in this role?

(e.g., <20 hours, 20–35 hours, 36–40 hours, 41+ hours)

**Experience with virtual care**

- How long have you been involved in delivering virtual care services? (e.g., <1 year, 1–3 years, 3–5 years, 5+ years)
- What prior experience, if any, did you have with delivering care virtually before joining Sydney Virtual Hospital?

**Domain: Knowledge**

(1) Can you share your experience taking a BPMH?

Can you describe the steps of the BPMH process at Sydney Virtual?

Prompt:

- What do you think is the role of BPMH?

**Domain: Professional role and identity**

(2) What contributions do you think nurses making towards the BPMH process?

What unique perspectives do you think nurses bring that differ from pharmacists’ or doctors’, if any?

What do you see as the advantages and or disadvantages nurses face taking on this process?

Prompt:

- Do you see it as part of your extended responsibility? If so, in what way?

**Domain: Emotion**

(3) When this BPMH practice change was first introduced in the virtual care setting, what concerns or reservations did you have initially?

Prompt:

- What were your initial thoughts about the BPMH program before the education and implementation? In its potential benefits and disadvantages at that stage?

**Domain: Education**

(4) What part of the BPMH education do you think were most helpful in preparing you for the actual process?

Prompt:

- What specific scenarios do you feel the education did not quite prepare you?
- Is there anything could be done differently? Any other changes you’d suggest for the education program?

**Domain: Beliefs about capabilities**

(5) How comfortable do you feel to collect a complete and accurate BPMH?

Prompt:

- What helps you feel more comfortable in the process?
- What specific action would you take after completing the BPMH, if any issues arise requiring follow-up?
- Have there been situations where you feel it is not clear what your actions should be?

**Domain: Environmental context and resources**

(6) What challenges have you experienced when performing BPMH?

Prompt:

- *If the participant cannot think of any, this prompt will then be used for guidance* For example, are there any internal and external factors such as time, workload, skill gaps, resource availability, patient interactions or access to medication information making the process more difficult?
- Any specific difficulties that come up relating to virtual care setting?
- How did you manage these?

**Domain: Environmental context and resources**

(7) In your view, what do you think Sydney Virtual Hospital is doing well when it comes to nurse-led BPMH?

Prompt:

- What do you think has helped make these successes possible?
- Are there any areas where you feel things could be improved and done differently?

**Domain: Feedback and support**

(8) Have you received any feedback or support about your BPMH practices? If so, did you find it constructive and helpful?

Prompt:

- Do you feel your efforts in conducting BPMH are valued and appreciated? Can you give an example in what way?
- What are some areas where you feel you could use additional support?

**Domain: Goals**

(9) In an ideal world, what would BPMH look like in the Virtual Care Centre in the near future?

- What do you think will help to get to that vision?

(10) Any final comments or suggestions you’d like to share regarding BPMH that we haven’t covered today?
